# Molecular optimization by capturing chemist’s intuition using deep neural networks

**DOI:** 10.1186/s13321-021-00497-0

**Published:** 2021-03-20

**Authors:** Jiazhen He, Huifang You, Emil Sandström, Eva Nittinger, Esben Jannik Bjerrum, Christian Tyrchan, Werngard Czechtizky, Ola Engkvist

**Affiliations:** 1 Discovery Sciences, R&D, AstraZeneca, Gothenburg, Sweden; 2 Medicinal Chemistry, Research and Early Development, Respiratory and Immunology (R&I), BioPharmaceuticals R&D, AstraZeneca, Gothenburg, Sweden; 3 Department of Pharmaceutical Biosciences, Uppsala University, Uppsala, Sweden; 4 Department of Mathematics and Mathematical Statistics, Umeå University, Umeå, Sweden

**Keywords:** Molecular optimization, Matched molecular pairs, Seq2seq, Transformer, Recurrent neural networks, ADMET

## Abstract

A main challenge in drug discovery is finding molecules with a desirable balance of multiple properties. Here, we focus on the task of molecular optimization, where the goal is to optimize a given starting molecule towards desirable properties. This task can be framed as a machine translation problem in natural language processing, where in our case, a molecule is translated into a molecule with optimized properties based on the SMILES representation. Typically, chemists would use their intuition to suggest chemical transformations for the starting molecule being optimized. A widely used strategy is the concept of matched molecular pairs where two molecules differ by a single transformation. We seek to capture the chemist’s intuition from matched molecular pairs using machine translation models. Specifically, the sequence-to-sequence model with attention mechanism, and the Transformer model are employed to generate molecules with desirable properties. As a proof of concept, three ADMET properties are optimized simultaneously: *logD*, *solubility*, and *clearance*, which are important properties of a drug. Since desirable properties often vary from project to project, the user-specified desirable property changes are incorporated into the input as an additional condition together with the starting molecules being optimized. Thus, the models can be guided to generate molecules satisfying the desirable properties. Additionally, we compare the two machine translation models based on the SMILES representation, with a graph-to-graph translation model HierG2G, which has shown the state-of-the-art performance in molecular optimization. Our results show that the Transformer can generate more molecules with desirable properties by making small modifications to the given starting molecules, which can be intuitive to chemists. A further enrichment of diverse molecules can be achieved by using an ensemble of models.

**Supplementary Information:**

The online version contains supplementary material available at 10.1186/s13321-021-00497-0.

## Introduction

A main challenge in drug discovery is finding molecules with desirable properties. A drug requires a balance of multiple properties, *e*.*g*. physicochemical properties, ADMET (absorption, distribution, metabolism, elimination and toxicity) properties, safety and potency against its target. To find such a drug in the extremely large chemical space (*i*.*e*. $10^{23}-10^{60}$) [1] is challenging. It is often that a promising molecule needs to be improved to achieve a balance of multiple properties. This problem is known as molecular optimization. Traditionally, chemists would use their knowledge, experience and intuition [2] to apply some chemical transformations to the promising molecule. In particular, the matched molecular pair (MMP) analysis [3, 4]—which compares the properties of two molecules that differ only by a single chemical transformation—has been widely used as a strategy by medicinal chemists to support molecular optimization [5–7]. Typically, similarity, transferability, and linear analoguing [8–10] are assumed and applied by the chemists to suggest transformations to improve the promising molecule. However, they are not generally true, and become more problematic and difficult to apply when optimizing multiple properties simultaneously.

To address these shortcomings, this work uses deep learning models to learn the transformations involved in molecular optimization directly from MMPs. Deep generative models, *e*.*g*. recurrent neural networks (RNNs) [11, 12], variational autoencoders (VAEs) [13–18], and generative adversarial networks (GANs) [19–22], coupled with reinforcement learning [19, 20, 22, 23], adversarial training [24–26], transfer learning [11], and different optimization techniques [13, 27], have been investigated to generate molecules towards desirable properties. Recently, chemical reactions are incorporated into neural networks for de novo design [28], which allows to generate synthesizable molecules with their synthesis routes. Additionally, conditional generative models [15, 18, 29, 30] have been developed where the desirable properties are incorporated as condition to directly control the generating process. Another approach is to use reinforcement learning to modify a molecule directly based on molecular graph representation [31, 32]. However, all the above methods are not direct and intuitive methods for molecular optimization. When given a promising molecule and the desirable properties, the direct way would be applying intuitive chemical transformations to achieve the desirable properties, while the above methods ignore the domain knowledge of chemical transformations.

In this paper, we focus on utilizing chemical transformations (*i*.*e*. MMPs), which reflect the chemist’s intuition to optimize a promising molecule. In particular, given a starting molecule and the desirable property changes, the goal is to generate molecules, which (i)have the desirable properties and (ii) are structurally similar to the starting molecule. As a proof of concept, we focus on optimizing ADMET properties which are applicable to all drug design projects. In particular, *logD*, *solubility* and *clearance* are optimized simultaneously, which are important properties of a drug. *LogD* measures the hydrophobicity of a molecule, which influences the molecule’s potency, metabolism and pharmacokinetic properties. *Solubility* influences absorption and bioavailability. *Clearance* is a measure of the capacity of drug removal by various organs, which is a key parameter to understand metabolic stability and dosing.

The problem of molecular optimization can be framed as a machine translation problem [33] in natural language processing (NLP), where a text is translated from one language to another. For molecular optimization, an input starting molecule is translated into a target molecule with optimized properties based on the simplified molecular-input line-entry system (SMILES) representation. The sequence-to-sequence (Seq2Seq) model [34] with attention mechanism [35] has been developed and applied in machine translation successfully. Recently, the Transformer, which only uses attention, has been shown to achieve the state-of-the-art (SOTA) performance in machine translation [36], and has become the basic building block of most SOTA architectures in NLP. Lately, it has been applied to predict chemical reactions and achieved SOTA performance (above 90% accuracy) on a common benchmark data set [37]. This motivated us to investigate the Seq2Seq with attention and the Transformer for molecular optimization tasks in this work.

The models are trained on MMPs extracted from ChEMBL. Since it is difficult to obtain the experimental property values for molecules in ChEMBL, we built a property prediction model for each ADMET property based on in-house experimental data. Then the models are applied on the extracted ChEMBL MMPs. In order to generate molecules towards customized desirable properties, the desirable property changes are concatenated with the source molecules’ SMILES, as input to the models.

The most relevant work to this paper are Jin et al. [24, 38, 39], who view molecular optimization as a graph-to-graph translation problem. A variational junction tree encoder-decoder (VJTNN) [24] was trained on a set of MMPs for molecular optimization. Based on VJTNN, Jin et al. [38] proposed a multi-resolution, hierarchically coupled encoder-decoder for graph-to-graph translation, and extended it to be conditioned on desirable property criteria, to allow for different user-specified property criteria and multi-property optimization. Recently, Jin et al. [39] proposed a new hierarchical graph encoder-decoder (HierG2G) by utilizing graph motifs as building blocks, which are frequently occurring substructures, to facilitate generating large molecules. It was also extended to graph-to-graph translation for molecular optimization, and outperformed VJTNN.

All the above models are based on molecular graph representations, while our models are based on SMILES representations and utilize the SOTA machine translation models, the Seq2Seq with attention and the Transformer. Although Jin et al. [24, 38, 39] compared their models with Seq2Seq and showed that they performed better, the Seq2Seq used only one one-layer long short-term memory (LSTM) in the encoder and decoder, while we use multiple layers. Additionally, the Transformer, has not been explored in molecular optimization. Therefore, we conduct a comparison over these three models, Seq2Seq with attention, Transformer and HierG2G. For HierG2G [39], the conditional extension in [38] is applied to support customized property optimization and multi-property optimization.

## Methods

### Molecule representation and property representation

**Fig. 1:**
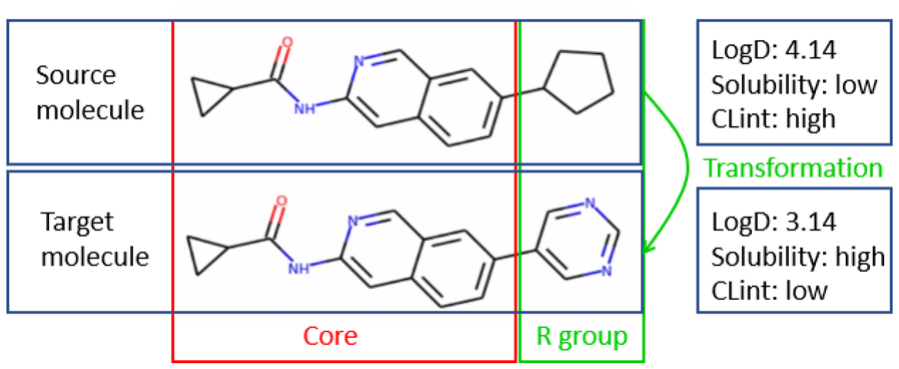
An example of a matched molecular pair and the properties

The models are trained on a set of MMPs together with the property changes between source and target molecules. Figure 1 shows an example of a MMP, and the properties of source and target molecules. The SMILES representation of molecules [40], as a string-based representation, is used in our study to facilitate the use of machine translation models from NLP.

**Fig. 2:**
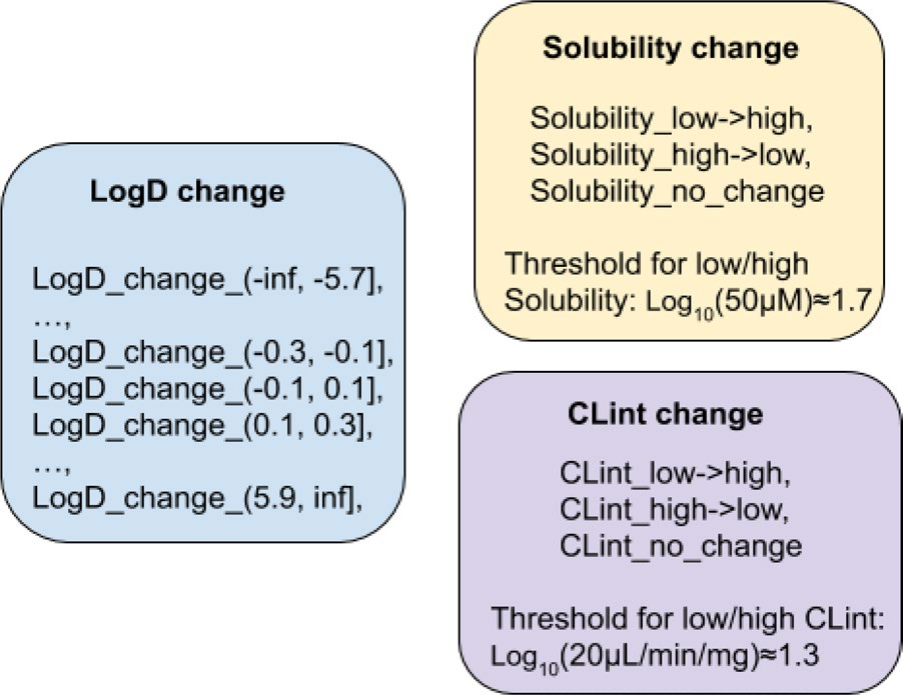
Property change encoding

Considering practical desirable criteria and experimental errors, *solubility* and *clearance* changes are encoded using three categories, while the change in *logD* is encoded into range intervals, with each interval length = 0.2 except for the two open intervals on the sides (Fig. 2). For *clearance*, human liver microsome intrinsic clearance (HLM CLint) is used in this work, and the thresholds for low/high *solubility* and low/high *CLint* are 50 $\mu$M and 20 $\mu$L/min/mg respectively (1.7 and 1.3 respectively in $log_{10}$ scale).

**Fig. 3:**
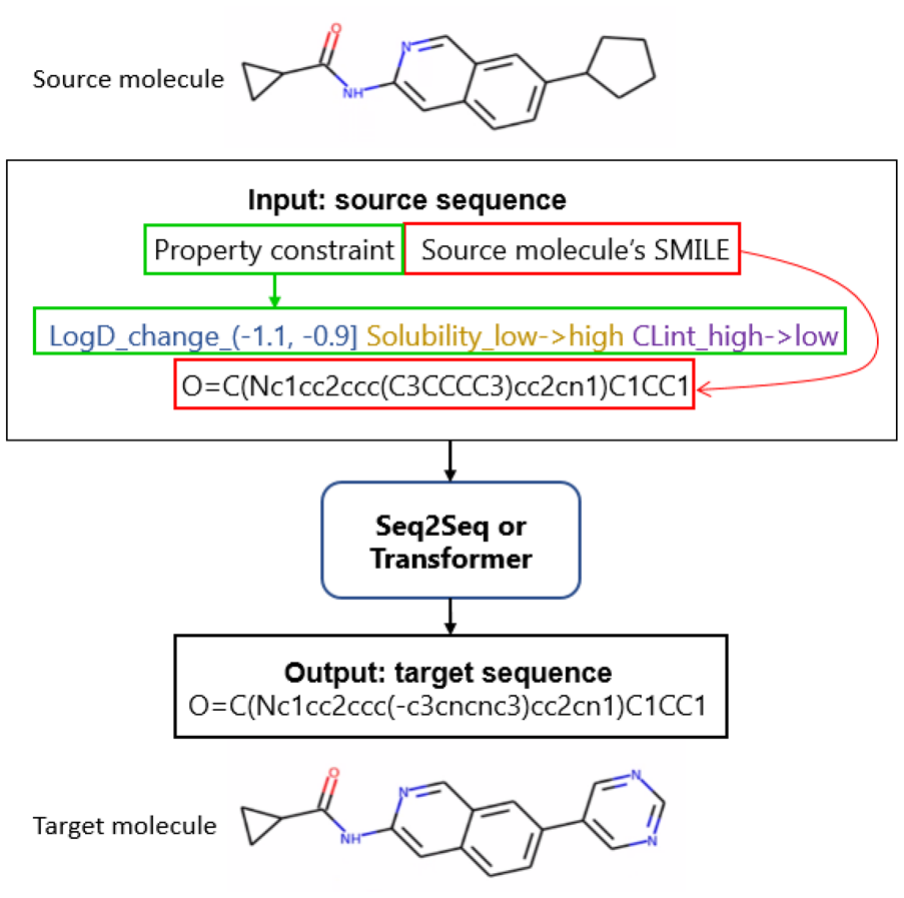
An example of source and target sequences fed into Seq2Seq or Transformer during training

In order to translate source molecules into target molecules with customized properties, the encoded property changes are concatenated with the SMILES representation of starting molecules as input sequences for machine translation models, while the target sequences are the SMILES representation of target molecules. Figure 3 shows an example of source and target sequences which are fed into machine translation models during training.

Given a set of MMPs $\{(X, Y, Z)\}$ where *X* represents source molecule, *Y* represents target molecule, and *Z* represents the property change between source molecule *X* and target molecule *Y*, the Seq2Seq with attention and the Transformer will learn a mapping $(X,Z)\in {\mathcal {X}}\times {\mathcal {Z}}\rightarrow Y\in {\mathcal {Y}}$ during training where ${\mathcal {X}}\times {\mathcal {Z}}$ represents the input space and ${\mathcal {Y}}$ represents the target space. During testing, given a new $(X,Z)\in {\mathcal {X}}\times {\mathcal {Z}}$, the models will be expected to generate a diverse set of target molecules with desirable properties.

### Seq2Seq with attention

The Seq2Seq [34] is a framework that maps an input sequence to an output sequence, which has wide applications, such as machine translation, text summarization, chatbot, question answering system, and image captioning. In particular, it has brought a major breakthrough in neural machine translation. The Seq2Seq is based on an encoder-decoder architecture using RNN. The encoder takes an input sequence, and compresses it into a context vector, defined by the hidden state in the last time step of encoder, which captures the information of the whole input sequence. Specifically, at each time step in the encoder, the RNN takes a word from the input sequence and a hidden state from the previous time step, and output a hidden state. The hidden state memorizes the words seen earlier and is updated at each time step, and the one from the last time step is called context vector, which captures the information of the whole input sequence. The context vector is then passed to the decoder to predict an output sequence. The drawback of the Seq2Seq is that it becomes difficult to deal with long sequence because the encoder has to compress the whole sequence into a single context vector in the last time step. To overcome this problem, attention mechanism [35] was introduced, which utilizes the hidden states at each time step from the encoder. It enables the decoder to focus on specific tokens in the input sequence when predicting each token in the output sequence.

In this paper, the Seq2Seq with attention is explored for molecular optimization (Seq2Seq refers to Seq2Seq with attention in the rest of this paper). First, all the source and target SMILES in our dataset were tokenized to construct a vocabulary, which contains all the possible tokens. Two special symbols were added, *start* and *end*, representing the start and end of a sequence respectively. In order to guide the model to generate molecules with different specified property constraints, the property changes between source and target molecule were concatenated with the source SMILES as the input sequence to Seq2Seq as illustrated in Fig. 3. Therefore, each possible single property change was treated as a token (*e*.*g*. LogD_change_(− 1.1, − 0.9]) and added to the vocabulary.

**Fig. 4:**
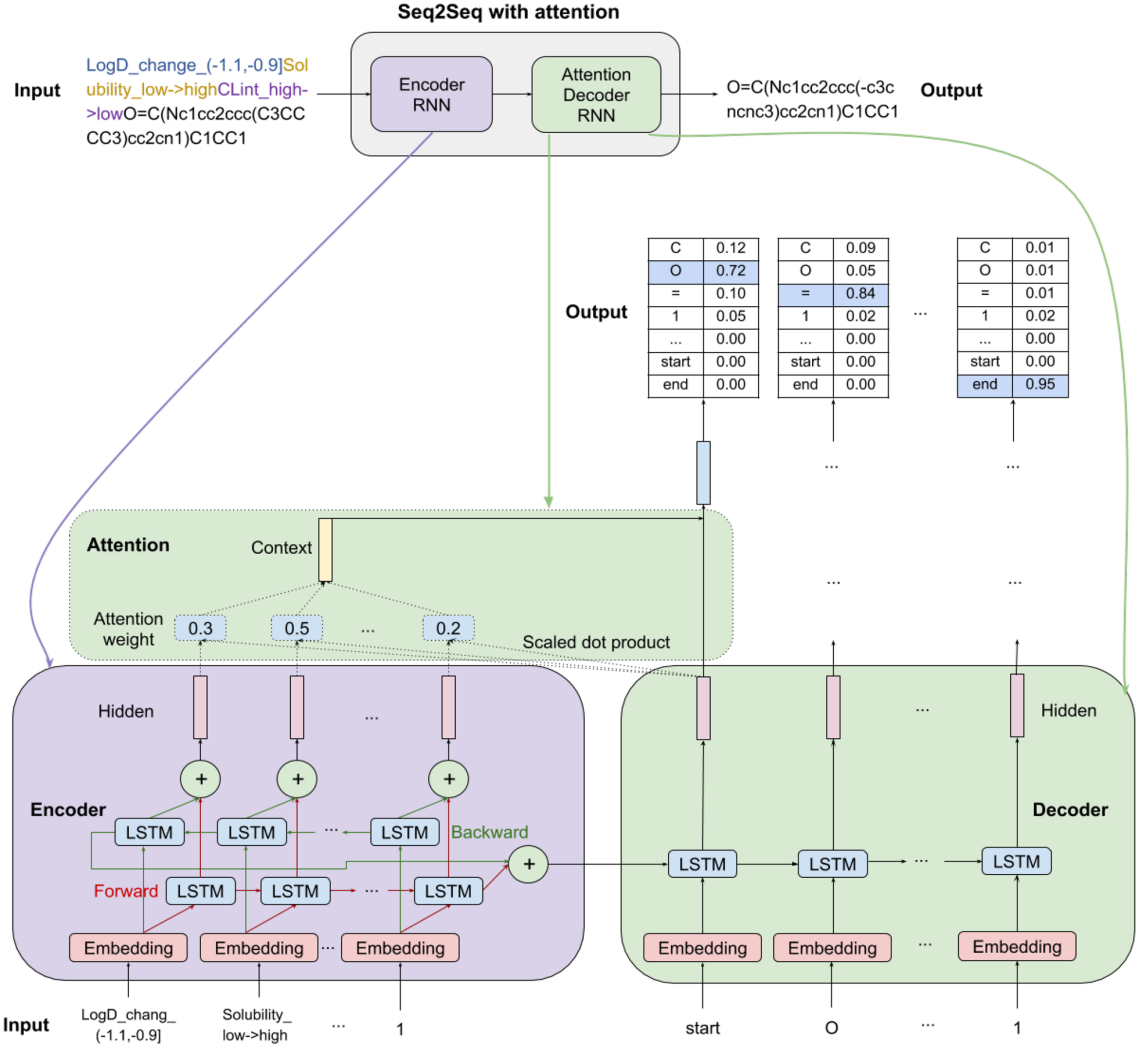
The Seq2Seq with attention architecture for molecular optimization

The model architecture is shown in Fig. 4. It consists of an encoder RNN and an attention decoder RNN, with LSTM cells. The encoder consists of an embedding layer of 256 dimensions and 5 stacked bidirectional LSTM layers with hidden size of 512 and dropout of 0.3. The embedding layer converts the input token at each time step into a continuous representation, which is then passed through the stacked bidirectional LSTM. At the last time step, the LSTM ouputs for both directions are summed and passed to the decoder. Similarly, the LSTM hidden states at each time step for both directions are summed and used to compute the attention with the hidden state from the current time step of decoder.

The decoder consists of an embedding layer of 256 dimensions and 5 stacked unidirectional LSTM layers with hidden size of 512 and dropout of 0.3. Additionally, it includes an attention layer. The initial input token is the *start* token. At each time step in decoding, the attention layer computes the attention weights which captures the importance of each source token for predicting the next target token. The attention weights are computed by the scaled dot product between the the hidden states at all time steps in the encoder and the hidden state at the current time step in the decoder, followed by a softmax function. Then a context vector is obtained by a weighted sum of the encoder hidden states at all time step. The context vector captures relevant information from every source token to help predict the next target token. It is concatenated with the hidden state at the current time step in decoder and then passed through a linear layer with a hyperbolic tangent activation function. The output is lastly passed through a linear layer to reshape to the vocabulary size, and a softmax activation function is applied to obtain the probabilities of each token in the vocabulary.

The model is trained to predict the next token of the target sequence, given previous tokens of the target sequence conditioned on the input sequence. In particular, teacher forcing, as a commonly used training technique for aiding efficient training of RNN, is used in the decoder, where the ground-truth target token in the training set at the current time step rather than the output generated by the model, is used as input for the next time step. Specifically, given a training set, $D=\{({\mathbf {x}}_{i}, {\mathbf {y}}_{i})\}$ where ${\mathbf {x}}_{i}$ and ${\mathbf {y}}_{i}$ represents the *i*th source sequence and target sequence respectively in the dataset *D*, we find ${\mathbf {\uptheta }}$ to minimize negative log likelihood (NLL):

1 $$\begin{aligned} \mathrm {NLL}({\mathbf {\uptheta }})=-\sum _{i \in D} \sum _{t=1}^{\left| {\mathbf {y}}_{i}\right| } \log {\text {P}}\left( y_{i,t} \mid {\mathbf {y}}_{i,1:t-1},{\mathbf {x}}_{i};{\mathbf {\uptheta }}\right) \end{aligned}$$

where ${\mathbf {\uptheta }}$ represents all the parameters in the model, $y_{i,t}$ represents the *t*th token of ${\mathbf {y}}_{i}$. After training, when using the model for generation, the ground-truth target sequence is not available and the output sequence is generated by sampling one token at a time from the distribution over the vocabulary until the *end* token is sampled. Specifically, multinomial sampling is used to generate multiple molecules for a given input sequence.

### Transformer architecture

**Fig. 5:**
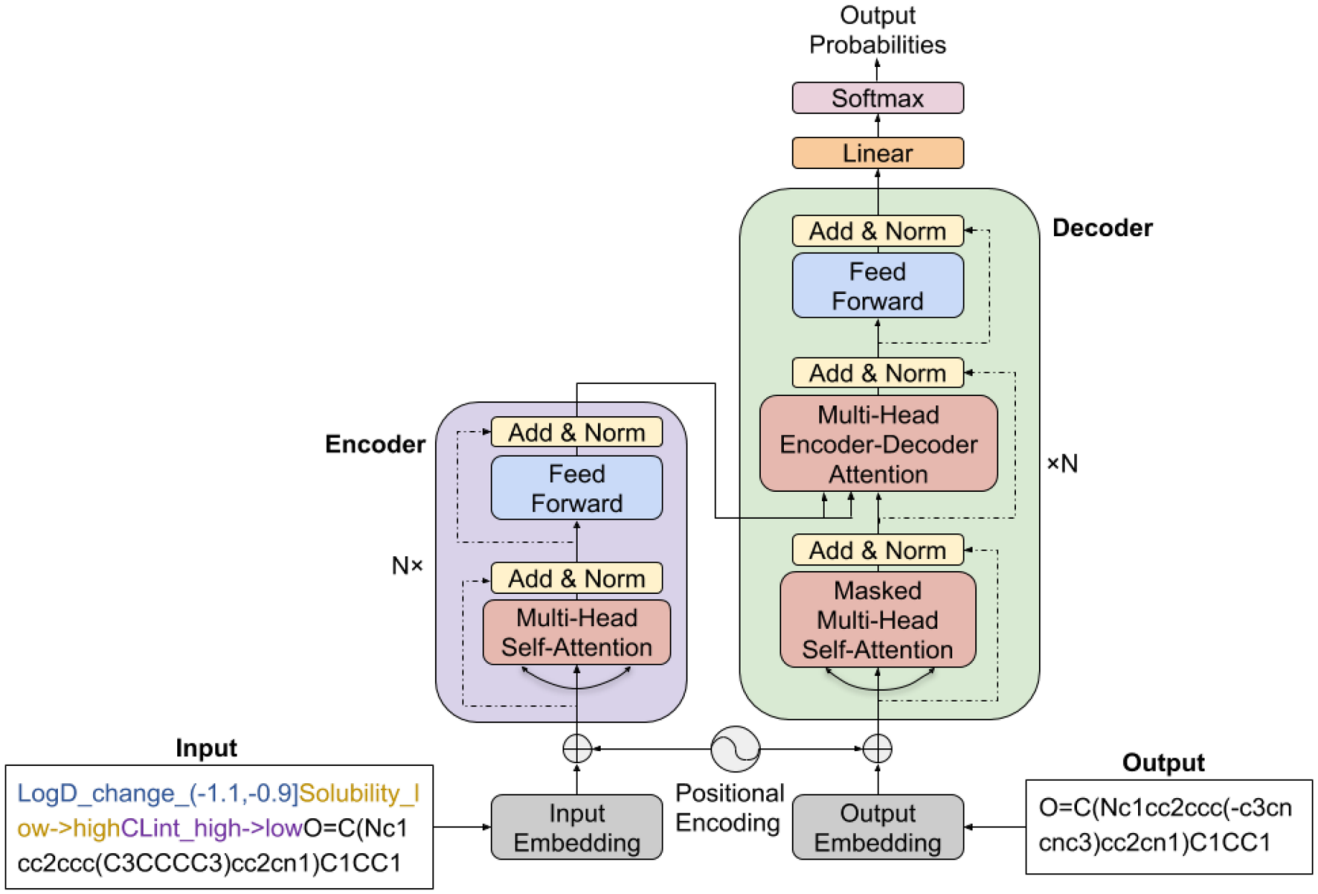
The Transformer architecture (following Vaswani et al. [36]) for molecular optimization

Although the Seq2Seq with attention has achieved great success in machine translation, it is still challenging to deal with long-range dependencies, and the sequential nature of the RNN prevents parallelization. The Transformer [36] was proposed to discard the RNN and rely only on attention mechanism instead. In this paper, the Transformer architecture illustrated graphically in [36] is explored for molecular optimization, as shown in Fig. 5. The Transformer consists of an encoder and a decoder. First, a vocabulary is constructed the same way as the Seq2Seq with attention. Before feeding the input sequence to the encoder, each token in the input sequence is converted into an embedding vector, followed by a positional encoding, which gives the embeddings order information.

*Encoder* The encoder consists of a stack of N identical encoder layers. Each encoder layer takes a list of input encodings from the previous encoder layer (list size is determined by the input sequence length) as input, and generates a list of output encodings that pass through the next layer. The input for the first encoder layer is the embedding of each token in the input sequence. Each encoder layer has two sub-layers: a multi-head self-attention sub-layer and a position-wise fully connected feed-forward network sub-layer. A residual connection is used around each of the two sub-layers, followed by layer normalization.

In the first encoder layer, for each positional token embedding at position *t* in the input sequence, it first flows through the multi-head self-attention sub-layer, which helps the encoder focus on relevant tokens in the input sequence to better encode it. Specifically, three vectors, Q (query), K (key), V (value) are first created from the input token embedding by multiplying with three weight matrices that are learned during training. These three vectors are used to compute the self-attention score for the input token at position *t*, which determines the importance of all the tokens in the input sentence for encoding the input token being processed. The score is computed by the scaled dot-product between Q of the input token at *t* being processed and K of each token in the input sequence, followed by a softmax function. Then a weighted sum of the value vectors of all tokens in the input sequence is obtained as the attention vector for the token at position *t* being processed. This process is for obtaining a single head attention. Multi-head attention [36] was introduced to help to focus on the input from different perspectives. Specifically, multiple weight matrices are learned to project the input embedding into multiple sets of Q, K, V, which are then used to derive multiple attention vectors. These attention vectors are then concatenated and projected to produce the final output of the multi-head self-attention sub-layer, which is then passed through the feed-forward neural network sub-layer. The output from the feed-forward neural network sub-layer is fed to the next encoder layer.

*Decoder* Similar to the encoder, the decoder consists of a stack of N identical decoder layers. Each decoder layer has three sub-layers, masked multi-head self-attention sub-layer, fully connected feed-forward network sub-layer, and encoder-decoder multi-head attention sub-layer. The decoder operates in a similar fashion to the encoder, except that the attention differ from those in encoder in the following: (i) while self-attention in encoder allows each position to attend all positions from previous encoder layer, self-attention in decoder only allows each position to attend earlier positions by masking the future positions. (ii) An additional encoder-decoder multi-head attention was introduced to help the decoder focus on specific parts of the input sequence, which is similar to the role of encoder-decoder attention mechanism in Seq2Seq with attention. Specifically, the output of the top encoder layer is transformed into a set of vectors K and V, which is used by each encoder-decoder multi-head attention sub-layer. Similar to Seq2Seq with attention, the model was trained using teacher forcing, and multinomial sampling was used to generate multiple molecules for a given input sequence.

### Model training and selection

The Seq2Seq training and inferencing was performed on a NVIDIA GeForce RTX 2080 Ti. The Adam optimizer with learning rate 0.0001 and a batch size of 128 were used. The hyperparameters were tuned based on previous experience [34, 36]. The Transformer training and inferencing was performed on a NVIDIA Tesla K80. The hyperparameters were tuned, and most remained the same as [36], except that the input and output encoding dimension was changed from 512 to 256, and label smoothing was changed from 0.1 to 0.

We saved a model at each epoch, and the best of these was chosen based on the accuracy on the validation set. The accuracy was computed by comparing the target molecule with one generated molecule using greedy decoding for each input sequence (property constraint and molecule) of the validation set. This is different from the evaluation with the test set. Here, we sample multiple molecules using multinomial sampling and the generated molecules are not necessarily required to be the same as the target molecule (if provided). The generated molecules need to satisfy the specified desirable properties and maintain structural similarity (enforced by the training) to the input molecules. The reasons of the different approaches are (i) it is time-consuming to generate multiple molecules for each sample of the validation set after each epoch during training (ii) and compute their properties to check if they satisfy the desirable properties after each epoch during training. (iii) During model optimization, we observed a tendency that the higher accuracy on the validation set, the more molecules with desirable properties generated on the test set.

### Data preparation

The models are trained on a set of MMPs extracted from ChEMBL together with the property changes between the source and target molecules. The properties (*logD*, *solubility* and *clearance*) are predicted from models built using the in-house experimental data.

#### Constructing matched molecular pairs

The matched molecular pairs (including reverse transformations) are extracted from ChEMBL using an open-source matched molecular pair tool [41]. All the molecules were standardized using MolVS [42]. There are 9,927,876 pairs considering the following constraints,

- The number of heavy atoms of the core ≤ 50
- The number of heavy atoms in R group ≤ 13
- The ratio of heavy atoms in the R group to the molecule R group ≤ 0.33
- The number of H-bond donors in R group ≤ 3
- The number of H-bond acceptors in R group ≤ 3
- AstraZeneca’s AZFilter=“CORE” [43] to filter out bad-quality compounds
- Each molecule’s property values are within 3 standard deviations of all molecules’ property values

2% from the full 9,927,876 pairs are randomly sampled for comparing three models because HierG2G does not scale well on large data. We randomly split it into 90% as training and validation, and 10% as test, and further split the 90% into 90% as training and 10% as validation, which results in 160,831 training, 17,871 validation and 19,856 test.

#### ADMET property prediction model

The property prediction models are built based on message passing neural network [44]. They are used for constructing data during training and also for evaluating the generated molecules during testing. In particular, in-house experimental data are used for building property prediction models. Table 1 shows the train and test size, root-mean-square error (RMSE), normalized RMSE (NRMSE) and $R^2$ for each property prediction model. More results on the experimental properties and predicted properties can be found in Figure S1 in Additional file 1.

**Table 1:** Property prediction model performance

	LogD	Solubility	HLM CLint
Train size	170,337	184,883	144,300
Train RMSE	0.304	0.485	0.264
Train NRMSE	0.041	0.079	0.083
Train $R^2$	0.935	0.774	0.749
Test size	18,927	20,543	16,034
Test RMSE	0.395	0.602	0.350
Test NRMSE	0.054	0.104	0.113
Test $R^2$	0.892	0.658	0.557

### Experimental settings

#### Test sets

Each test sample (*X*, *Z*) consists of two parts, the starting molecule being optimized *X* and the desirable property change *Z*, which therefore determines the input data space ${\mathcal {X}}$. In order to evaluate our models comprehensively, three test sets are constructed:

- *Test-Original* is the original test set $\{(X,Z)_{test}\}$ (10% of dataset) with 19,856 samples, where the desirable property changes are determined by the MMPs in the test set. It has the same input space as the training set, which is typical in machine learning models. But note that each test sample $(X,Z)_{test}$ has not been unseen in the training set. This test set is used to test if our models can generalize well on unseen different combinations of *X* and *Z* in the input space ${\mathcal {X}}\times {\mathcal {Z}}$.
- *Test-Molecule* is a subset of Test-Original with 12,721 samples where the starting molecules are not seen in the training set (by comparing their canonical smiles to be identical), $\{(X,Z)_{test}|X\notin {\mathcal {X}}_{train}\}$ where ${\mathcal {X}}_{train}$ represents the set of source molecules in the training set. This set is used to test if our models can generalize well on unseen (*X*, *Z*) with further constraint of unseen starting molecules.
- *Test-Property* consists of 7,813 starting molecules with low *solubility*, high *CLint*, and *logD* between 2 and 4.4 in Test-Original. For the logD change, while the exact task is performed on Test-Original and Test-Molecule, here we require that the target logD property has to be (i) lower than the starting value and (ii) in range of 1.0-3.4. This is considered to be the desirable range optimization, as reflected in the in-house data distribution. Overall, we are interested in optimizing all the starting molecules to achieve lower *logD* (constrained to 1.0-3.4), high *solubility* and low *CLint*. The desirable property change for all starting molecules is set to LogD_change_=(-1.1,-0.9], Solubility_low$\rightarrow$high, CLint_high$\rightarrow$low. Therefore, the test set can be represented by $\{ (X,Z\}|(X\in {\mathcal {X}}_{test}) \wedge (\textit{solubility}(X)=low) \wedge (CLint(X)=high) \wedge (\textit{logD}(X)\in [2.0, 4.4])\wedge (Z=LogD\_change\_($- 1.1, - $0.9]\, Solubility\_low\rightarrow high CLint\_high\rightarrow low)\}$. This test set is used to test if our model can generalize well on a particular property change we are interested in.

#### Evaluation metrics

Aligning with our goal, the models are evaluated in two main aspects,

- *Satisfying all three desirable properties* For each starting molecule in the test set, 10 unique valid molecules, which are not the same as the starting molecule, were generated, and the number of molecules satisfying all three desirable properties out of the 10 generated molecules was counted. The ADMET property prediction model described earlier is used to compute the properties of generated molecules. Additionally, the model error (Test RMSE) in Table 1 is considered to determine if a generated molecule satisfies its desirable properties. For *logD*, the generated molecules with $|{{logD}}_{generated}-{{logD}}_{target}|\le 0.4$ will be considered as satisfying desirable *logD* constraint. For *solubility*, the threshold for low and high will be a range considering the model error, *i*.*e*. 1.7±0.6. The generated molecules with ${{solubility}}\le 2.3$ will be considered as low, and those with ${{solubility}}\ge 1.1$ will be considered as high. Similarly, for *CLint*, the threshold is 1.3±0.35.
- *Generation of MMPs* The MMP analysis was performed on the starting molecules and generated molecules to check if the generated molecules have single transformation to the starting molecules. Furthermore, the ratio of heavy atoms in the R group to the generated molecule ${R_{group}}\leq0.33$ and $R_{group}\leq0.50$ are examined.

#### Baseline

We compare our models, Seq2Seq and Transformer with the following baselines,

- *HierG2G* HierG2G training and inferencing was performed on a NVIDIA Tesla V100. The hyperparameters were tuned, and most remained the same as [39], except that *beta* was changed from 0.3 to 0.6.
- *MMP* A natural comparison would be exhaustively applying all MMP transformation rules to the starting molecules and selecting those yielding the specified desirable properties. However, a combinatorial issue occurs when applying all substructure search operations to all molecules and calculating properties on all outcomes. Therefore, we adopted a simplified baseline: we assume that we already know the R group to be substituted in the starting molecule, and randomly selected 10 R groups from all the possible R groups for substitution. The number of molecules satisfying all three desirable properties out of the 10 resulted molecules was reported. This allows for a direct comparison with our models where 10 molecules were generated as well.

#### K-sample Anderson–Darling test

The *K*-Sample Anderson–Darling Test [45] is a nonparametric test for testing if *k*-samples are drawn from the same population without having to specify the distribution function of that population. It is applicable to continuous and discrete data. This test is used to compare different models’ performance in terms of satisfying all three desirable properties.

## Results and discussion

### Data statistics

**Fig. 6:**
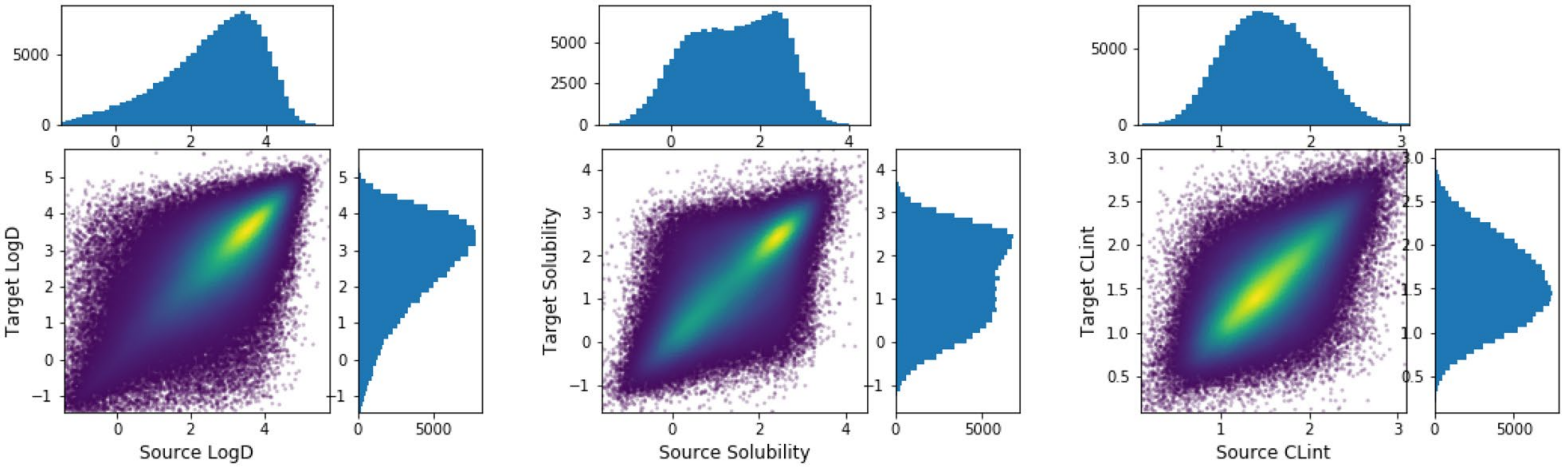
Source vs. target molecule’s properties and their distributions on the training set (*solubility* and *CLint* are in $log_{10}$ scale)

**Fig. 7:**
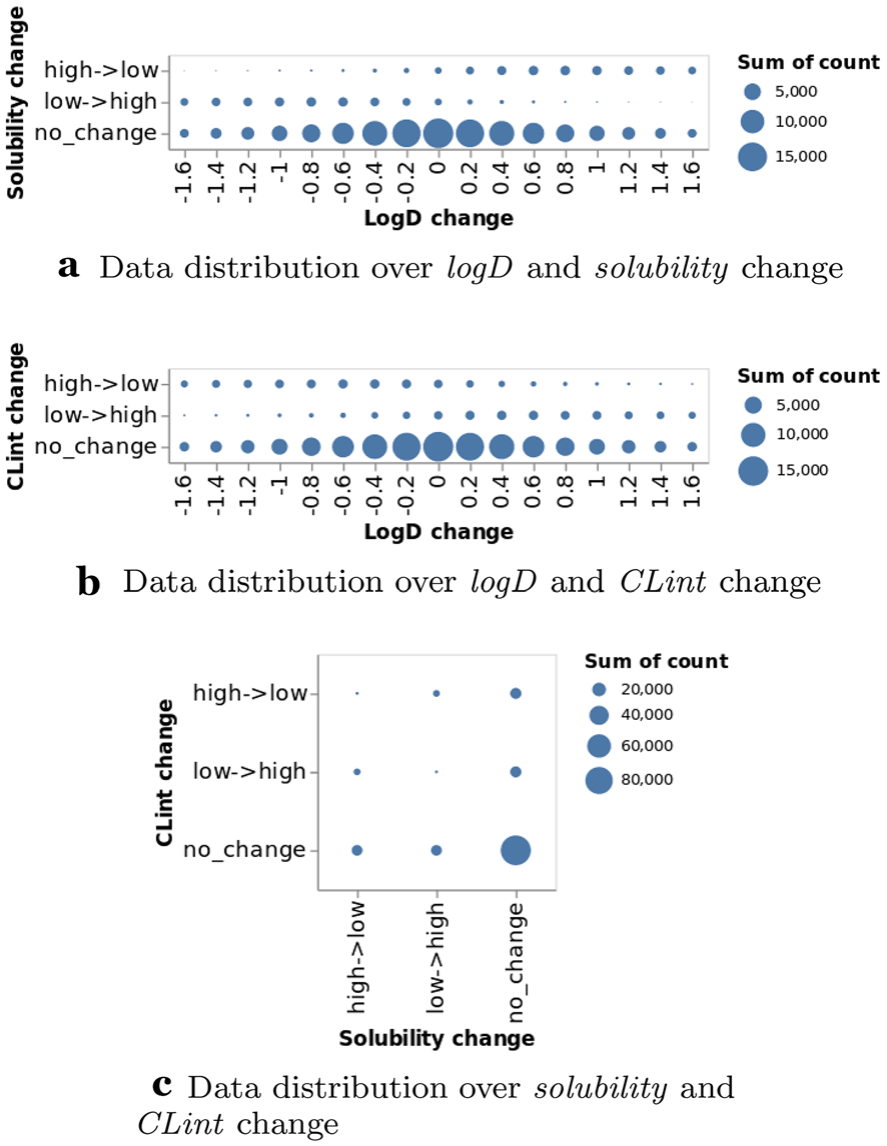
Data distribution over pairwise property changes on the training set where the circle size corresponds to counts. In **a**, **b** each tick *x* in horizontal axis represents $(x-0.1, x+0.1]$, *e*.*g*. 0 represents $(-\,0.1, 0.1]$. For ease of presentation, $x<-1.6$ and $x>1.6$ are not shown here

Figure 6 shows the source and target molecule’s properties distribution on the training set. The property distribution for the source molecules is the same as the one for the target molecules because reverse transformations are included in the data set. Figure 7 shows the training data distribution over pairwise property changes where most MMPs result in no change in properties. However, such MMPs are still useful because we could generate molecules with same properties, but different structures. It would also be useful when it is desired to keep some properties unchanged and change some other properties. Additionally, it can be seen from Fig. 7a, b that *solubility* tends to be negatively correlated with *logD*, and *CLint* tends to be positively correlated with *logD*.

Figure 10a shows the top 20 most frequently occurring transformations on the training set, which are encoded as SMIRKS [41]. The most frequently occurring transformation is [*:1][H]$>>$[*:1]C where a hydrogen is replaced by a methyl group and its reverse transformation. Note that they do not occur with exactly same frequency because we sampled 2% from the full MMPs. Table 2 shows the statistics of transformations on the training set where around 51.9% of transformations only occur once. The top 20 most frequently occurring transformation only accounts for around 8.2% of the training set, which means there are no dominant transformations.

**Table 2:** Transformation statistics on the training set

Percentage of unique transformations	59.4%
Percentage of transformations that occur only once	51.9%
Percentage of the most frequently occurring transformation	1.2%
Percentage of the top 20 most frequently occurring transformations	8.2%

### Unconditional models vs. conditional models

**Fig. 8:**
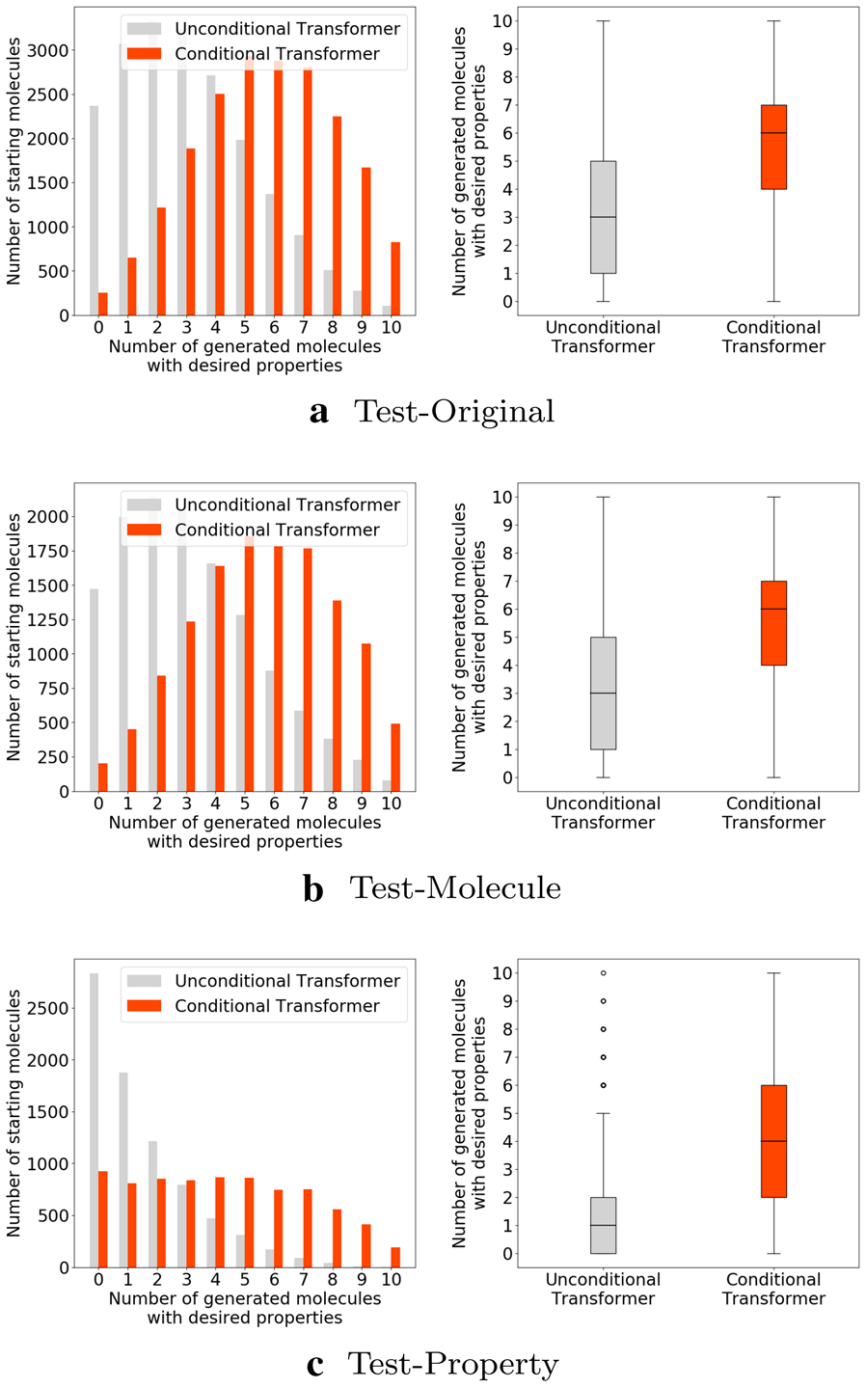
Number of generated molecules with desirable properties per molecule using unconditional Transformer and conditional Transformer on three test sets. Conditional Transformer outperforms unconditional Transformer using K-Sample Anderson–Darling Test at significance level of 0.1%

This set of experiment compares conditional models—which use source molecule and property criteria as input, with unconditional models—which use only source molecule as input. For each starting molecule in the test set, 10 unique valid molecules, which are not the same as the starting molecules, were generated. Figure 8 shows the performance of unconditional Transformer and conditional Transformer in terms of satisfying all three desirable properties on three test sets. K-Sample Anderson–Darling Test was performed, and conditional Transformer was found to statistically outperform unconditional Transformer. In particular, 50% of the starting molecules in Test-Original and Test-Molecule have at least 6 molecules with desirable properties out of the 10 generated molecules using conditional Transformer, while the number dropped to 3 using unconditional Transformer. Similar results have been found on the comparison of unconditional Seq2Seq and conditional Seq2Seq (Additional file 1: Figure S2).

#### Discussion

*Why does conditional models perform better than unconditional models?* It is shown that using property criteria as additional input can help generating molecules with desirable properties. One reason is that unconditional models are trained only on MMPs (*X*, *Y*) without property changes *Z*, with only source molecule *X* as input and target molecule *Y* as output. In this case, given a source molecule, it can be mapped to different target molecules with different properties because there could be multiple target molecules that are MMP with the source molecule in the training set. However, when using conditional models, the property change *Z* between source molecule and target molecule is used as part of input, which guides the model to generate molecules with desirable properties.

*What do the results on three test sets convey?* The performance on Test-Original shows that conditional models can generalize well on the unseen combination of starting molecules and desirable property changes, while the performance on Test-Molecule shows conditional models can generalize well on the combination of unseen starting molecules and seen/unseen property changes. On Test-Property, both unconditional and conditional models perform worse compared with the ones on Test-Original and Test-Molecule, especially unconditional models. Note that Test-Property is challenging because only 344 (0.2%) samples out of 160,831 training samples have the property change of interest.

### Model comparison for conditional models

This set of experiment mainly compares the conditional version of the three models: Seq2Seq, Transformer and HierG2G.

#### Satisfying multiple desirable properties

**Fig. 9:**
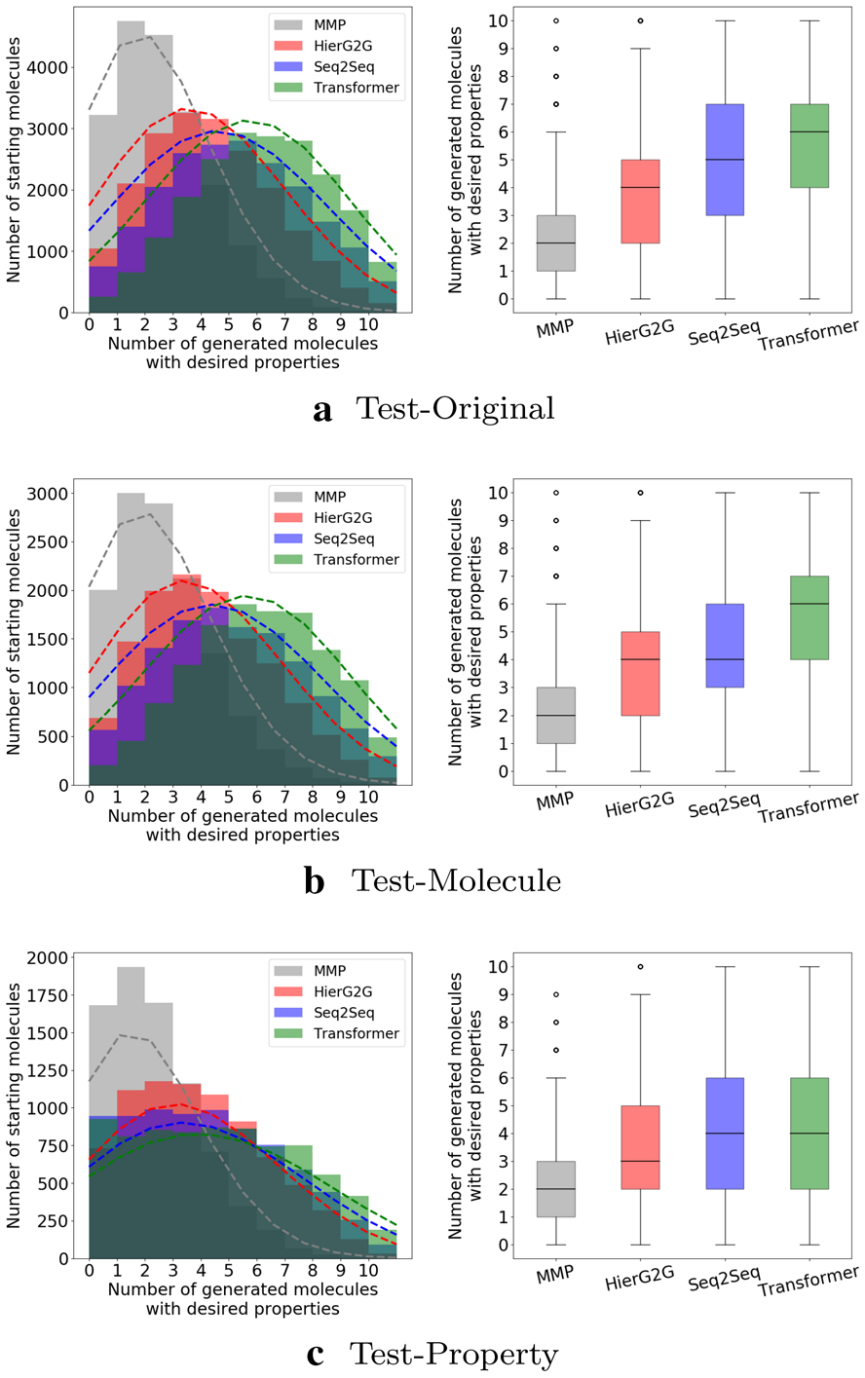
Number of molecules with desirable properties among 10 generated molecules per molecule using conditional version of HierG2G, Seq2Seq and Transformer on three test sets. The difference between each two is significant using K-Sample Anderson–Darling test at significance level of 0.1%

Figure 9 compares the performance of the Seq2Seq, the Transformer, HierG2G and the MMP baseline in terms of satisfying all three desirable properties on three test sets. The Transformer performs best, with more generated molecules satisfying desirable properties.

#### Discussion

*Comparison with MMP baseline* The MMP baseline performs much worse since we randomly selected 10 R groups as we discussed in section Baseline. While it is highly computational demanding to apply all MMP transformation rules, rank and find the ones yielding desirable properties, our model can directly generate molecules with desirable properties. Additionally, the generated molecules are not restricted to be known MMPs (Table 3). Our model could in principle allow for several modifications as well as modifications not corresponding to MMPs if intended (not topic of this publication).

**Table 3:** Comparison of the percentage of generated molecules from HierG2G, Seq2Seq and Transformer that are MMPs with starting molecules

	HierG2G			Seq2Seq			Transformer		
	MMP_0.33 (%)	MMP_0.50 (%)	in Train (%)	MMP_0.33 (%)	MMP_0.50 (%)	in Train (%)	MMP_0.33 (%)	MMP_0.50 (%)	in Train (%)
Test-Original	52.50	61.19	45.46	73.55	83.32	43.59	*90.45*	*96.49*	48.69
Test-Molecule	25.87	30.98	54.99	67.87	78.48	51.90	*88.83*	*95.58*	56.64
Test-Property	13.04	15.20	41.49	72.32	81.77	36.35	*90.69*	*96.37*	42.02

Among *all the generated molecules* for each test set, *MMP_0.33* and *MMP_0.50* represent the percentage of generated molecules that are MMPs with their corresponding starting molecules and the ratio of heavy atoms in R group to the generated molecule $R_{group}\leq0.33$ and $R_{group}\leq0.50$ respectively. Among all the transformations results from MMP_0.33, *in Train* represents the percentage of transformations that are seen in the training set

For MMP_0.33 and MMP_0.50, higher values are better, and the best values are in italics

*Benchmarking* The two main benchmarking suites in the field of de novo molecular generation: MOSES [46] and GuacaMol [47] are not directly comparable and suitable here since they are designed for benchmarking the generative models which generates molecules from scratch, while we focus on improving a given input molecule towards its specified desirable properties. The improved molecules are required to have small modifications to the input molecule, which is enforced by training on the MMPs. This was not directly tackled by the generative models which are trained on a set of molecules of interest.

#### Generation of MMPs

For each starting molecule in the test set and the 10 generated molecules, MMP analysis [41] was performed between the starting molecule and each generated molecule. Table 3 shows the percentage of generated molecules that are MMPs with their corresponding starting molecules and the ratio of heavy atoms in R group to the generated molecule $R_{group}\leq0.33$ and $R_{group}\leq0.50$. Additionally, among all the transformations results from $R_{group}\leq0.33$, the percentage of transformations that are seen in the training set is reported. It can be seen that the Transformer generates much more MMPs than the Seq2Seq and HierG2G for all the three test sets.

*Discussion* The Transformer generated much more molecules with single transformation to the starting molecules. This mimics the chemist’s strategy that applying single transformations when optimizing a starting molecule. Additionally, looking at “in Train”, all three models have learned to use not only the existing transformations in the training set but also novel transformations that have not been seen in the training set, to optimize novel combinations of starting molecules and specified desirable properties. Note that the MMP concept is used as a general concept for capturing the chemist’s intuition. This does not imply that the MMP concept is the only viable and solely strategy applied, but nevertheless due to its simplicity of linear analoguing it is commonly used. Furthermore, there are several well established algorithms [41] to access and analyze MMPs readily supporting structure-property relationship analysis.

**Fig. 10:**
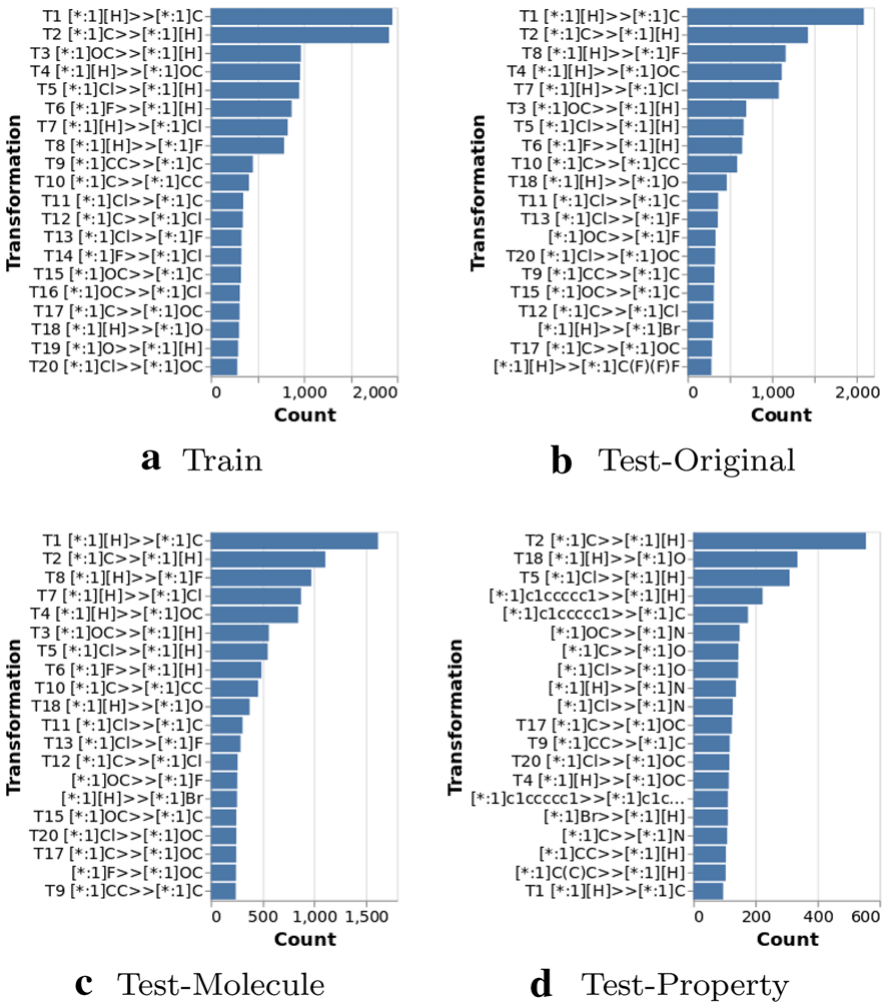
Top 20 most frequently occurring transformations on **a** Training set, and the top 20 most frequently occurring transformations among generated molecules from Transformer on **b** Test-Original, **c** Test-Molecule and **d** Test-Property

*Top 20 most frequently occurring transformations generated by Transformer* We further check the top 20 most frequently occurring transformations among the generated molecules from the Transformer, and compare them with the ones on the training set, as shown in Fig. 10. Most of the generated transformations on Test-Original and Test-Molecule are very similar to the ones on the training set.

*Discussion* The result indicates that the Transformer model has captured the transformations on the training set. The generated transformations on Test-Property are more different from the ones on the training set compared with the other two test sets. It is reasonable because the input space on Test-Property is very different from the one on the training set due to the constraint of particular property change. The generated transformations on Test-Property are biased towards generating molecules with that particular property change.

#### MMPs within generated desirable molecules

We take a closer look at the generated molecules with desirable properties from three models, and examine the percentage of MMPs (Table 4). The Transformer can generate much more desirable molecules than HierG2G and Seq2Seq, with 8–18%, and 4–12% absolute improvement respectively. Within the generated molecules with desirable properties, above 90% of the desirable molecules generated from the Transformer make single transformation to starting molecules and the ratio of the change (R group) compared to the generated molecule is no more than 1/3, while the number dropped significantly to around 55% and 74% for HierG2G and Seq2Seq respectively. Clearly, the Transformer can generate molecules with desirable properties by making small modifications to starting molecules as a chemist would do.

**Table 4:** Comparison of the percentage of generated *desirable* molecules from HierG2G, Seq2Seq and Transformer that are MMPs with starting molecules

	HierG2G			Seq2Seq			Transformer		
	Desirable (%)	MMP_ 0.33_D (%) (%)	MMP_ 0.50_D (%)	Desirable (%)	MMP_ 0.33_D (%)	MMP_ 0.50_D (%)	Desirable (%)	MMP_ 0.33_D (%)	MMP_ 0.50_D (%)
Test-Original	38.66	62.94	70.77	46.94	79.66	88.11	*56.14*	*92.67*	*97.64*
Test-Molecule	37.62	56.81	65.44	45.22	74.81	84.26	*56.74*	*91.41*	*96.98*
Test-Property	33.58	55.70	63.28	37.67	74.32	83.43	*41.75*	*91.06*	*96.83*

*Desirable* represents the percentage of molecules with desirable properties among all the generated molecules. Among *all these generated molecules with desirable properties* for each test set, *MMP_0.33_D* and *MMP_0.50_D* represent the percentage of generated molecules that are MMPs with their corresponding starting molecules and the ratio of heavy atoms in R group to the generated molecule $R_{group}\leq0.33$ and $R_{group}\leq0.50$ respectively

The results in italics indicate the best values; higher values are better

#### Validity

We sampled 10 molecules for each sample on the test set, and reported the percentage of valid molecules (by RDKit) among all the generated molecules (Table 5). All of the models have achieved good validity, with Seq2Seq around 95%, Transformer 98% and HierG2G 99%.

**Table 5:** The percentage of valid molecules among all the generated molecules on the test set

	HierG2G (%)	Seq2Seq (%)	Transformer (%)
Test-Original	99.98	95.00	98.50
Test-Molecule	99.98	94.60	98.25
Test-Property	99.99	95.54	98.55

### Varying performance of three models

Although the Transformer outperforms the Seq2Seq and HierG2G overall, it is not clear if it performs best for each test starting molecule. Therefore, we are interested in the following questions, which will help us understand if it will be beneficial to use all three models together.

1. Does the Transformer always perform best for each starting molecules in the test set?
2. Are the generated molecules from the three models the same or different?

**Fig. 11:**
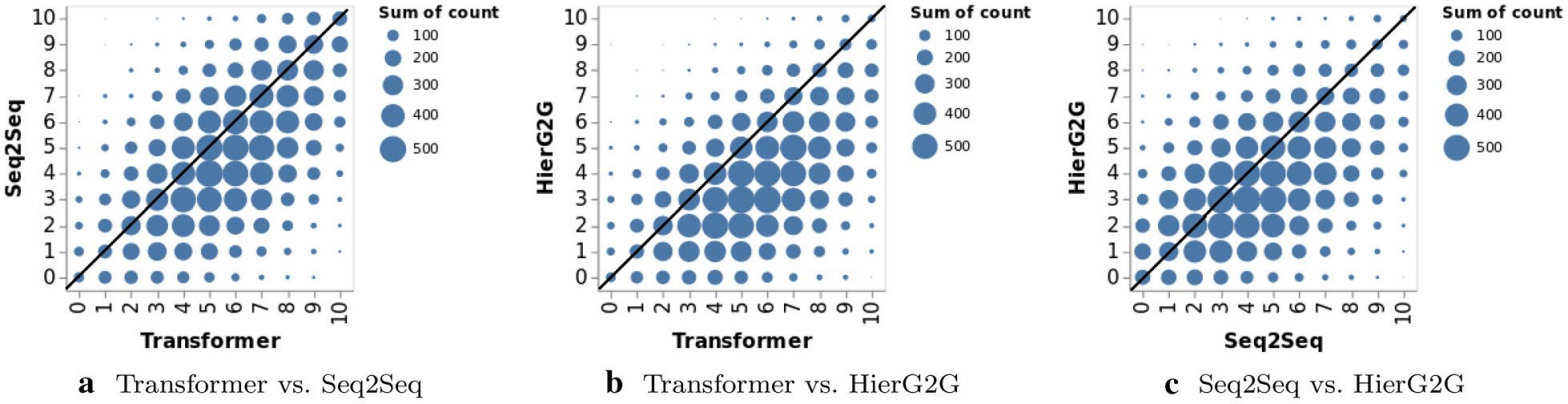
Pairwise comparison of HierG2G, Seq2Seq and Transformer on the number of molecules with desirable properties out of 10 generated molecules per molecule on Test-Original. The x axis and y axis represent the number of molecules with desirable properties out of 10 generated molecules. If two models always generate the same number of desirable molecules for each same starting molecule, all points will lie on the diagonal line

To answer the first question, we examine if one model always generates more desirable molecules for each starting molecule than the other two models. Figure 11 shows the pairwise comparison of three models on the number of molecules with desirable properties out of 10 generated molecules on Test-Original. The Transformer generated more desirable molecules than the Seq2Seq and HierG2G for most starting molecules, with those dots lie below the diagonal line in Fig. 11a, b. However, there are still some starting molecules where the Transformer generated less desirable molecules. Table 6 shows the percentage of starting molecules for which each model generates either less or more desirable molecules compared with the other two models on Test-Original. For 46.20% of the starting molecules on Test-Original, the Transformer generated more desirable molecules than HierG2G and Seq2Seq. But there are 10.02% + 21.81% = 31.83% of the starting molecules, where either HierG2G or Seq2Seq generated more desirable molecules. Therefore, it could be beneficial to use all three models together.

**Table 6:** Among all the starting molecules in Test-Original, the percentage of starting molecules for which each model generates either less or more desirable molecules when compared with the other two models

	Less than the others (%)	More than the others (%)
HierG2G	46.50	10.02
Seq2Seq	23.16	21.81
Transformer	8.89	46.20

**Fig. 12:**
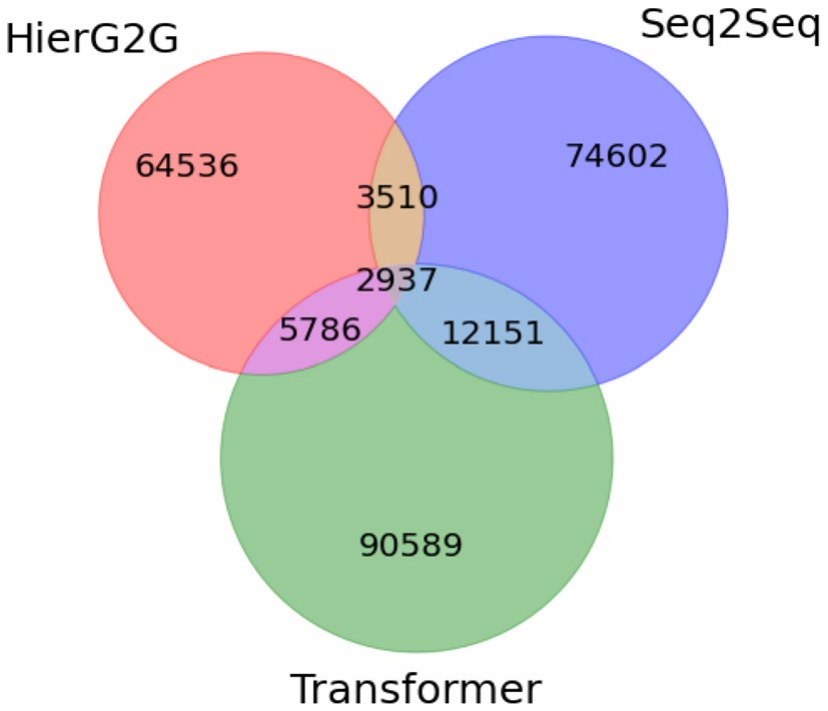
The overlap of the generated molecules with desirable properties from HierG2G, Seq2Seq and Transformer given each starting molecule in Test-Original. Each circle represents the set of molecules with desirable properties for each model. Transformer generated more desirable molecules than HierG2G and Seq2Seq, with the biggest circle. Most generated molecules with desirable properties from three models given the same starting molecule are different from each other, with only 2,937 identical molecules. Transformer and Seq2Seq have more identical desirable molecules (12,151) compared with (HierG2G, Seq2Seq) and (Transformer, HierG2G)

The second question was examined to see if the three models can generate diverse desirable molecules. For each starting molecule in Test-Original, we compute the overlapping and non-overlapping set of generated desirable molecules from HierG2G, Seq2Seq and Transformer, and sum the numbers over all starting molecules, which results in the Venn diagram in Fig. 12. First, the Transformer generated more desirable molecules than the Seq2Seq and HierG2G. Second, there is not much overlap of the generated desirable molecules among the three models. Third, the Transformer and Seq2Seq generate identical desirable molecules more frequently than the other two pairs. The reason might be that the Transformer and Seq2Seq are both based on SMILES representation and have a more similar working mechanism compared to HierG2G which is based on graph representation. Overall, the three models can generate diversified sets of molecules with desirable properties, which encourages us to use them in an ensemble way to enrich the generated desirable molecules.

**Fig. 13:**
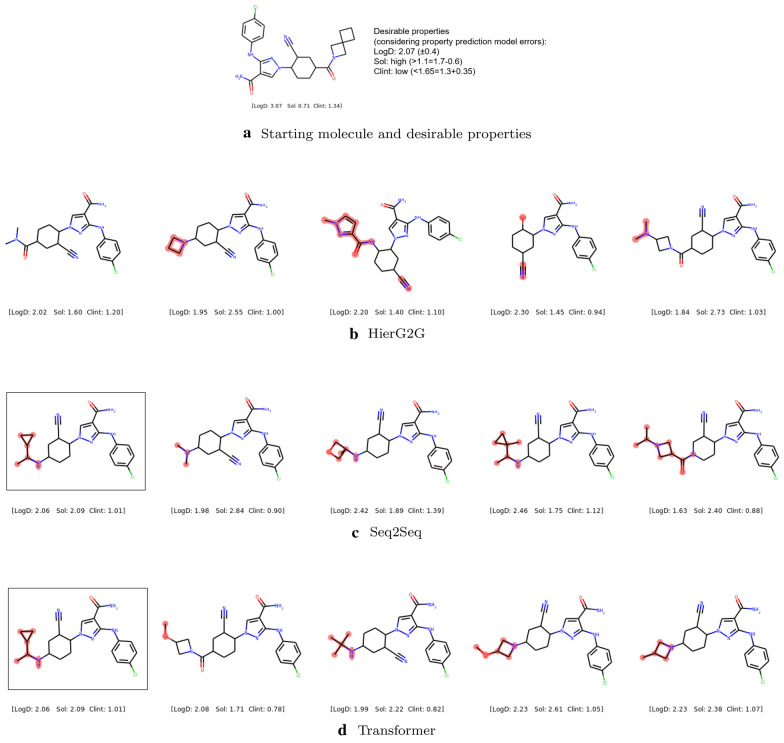
Example of diverse molecules with **a** desirable properties generated by **b** HierG2G, **c** Seq2Seq and **d** Transformer. The changes in the generated molecules compared with starting molecule are highlighted in red. Seq2Seq and Transformer have one molecule overlapping outlined by black rectangle

Figure 13 shows an example of the diverse molecules with desirable properties generated by HierG2G, Seq2Seq and Transformer. Given the starting molecule [48] and desirable properties in Fig. 13a, the three models generate diverse molecules with desirable properties and different small modifications to the starting molecule. The Seq2Seq and the Transformer have one molecule overlapping as outlined by the black rectangles. Overall, we see the diverse set of desirable molecules that the three models generated.

## Conclusions and future work

The molecular optimization problem was framed as a machine translation problem where a given molecule is translated into a molecule with optimized properties based on the SMILES representation. Two machine translation models, the Seq2Seq with attention and the Transformer have been investigated to generate molecules with desirable properties by capturing the chemist’s intuition, *i*.*e*. MMPs. The property changes have been incorporated into the input (starting molecule being optimized) as condition to guide the models to generate molecules with different combinations of property constraints. Given a starting molecule and user-specified properties, our models can generate molecules satisfying multiple property constraints while maintaining the structural similarity to the starting molecule. Specifically, for the Transformer, around half of all the generated molecules satisfied all target properties, and within the generated molecules with desirable properties, around 90% had a single transformation with respect to the starting molecules and no more than 1/3 change.

This can be beneficial to lead optimization, where a promising molecule needs to be improved to achieve a balance of multiple properties. The small modifications to the starting molecule, which mimic the chemist’s strategy, would be intuitive for chemists and provide insights for designing new molecules. Our deep learning models start from capturing the chemist’s intuition from MMPs, but they go beyond the working assumptions of chemists, e.g. transferability where the effect of a chemical transformation is assumed to be generalized to different molecular context. As data-driven approaches, our models can learn the intuitive chemical transformations without explicit assumptions.

The Seq2Seq and Transformer, were compared to HierG2G, a graph-to-graph translation model for molecular optimization. The Transformer performed best overall in generating more molecules with desirable properties and structurally similar to starting molecules. However, Seq2Seq and HierG2G can still generate different molecules with desirable properties. We believe the ensemble use of three models will contribute to a further enrichment of diverse molecules.

We have extracted a dataset of MMPs from ChEMBL with the source and target molecules’ ADMET properties (*i*.*e*. *logD*, *solubility* and *clearance*) predicted by the property prediction models trained on a large number of in-house experimental data. We believe it will be beneficial for the studies of MMP analysis and optimizing ADMET properties.

As a proof-of-concept, we have focused on optimizing ADMET properties since they are general, important and applicable to all drug design projects. In principle, our model can be trained to optimize other properties as well, *e*.*g*. synthetic accessibility and bioactivity.

## Supplementary Information

**Additional file 1. ** Additional figures.

## Data Availability

All source code and datasets used to produce the reported results can be found at https://github.com/MolecularAI/deep-molecular-optimization.

## Abbreviations

SMILES: Simplified Molecular-Input Line-Entry System
Seq2Seq: Sequence to sequence
SOTA: State-of-the-art
ADMET: Absorption, distribution, metabolism, elimination and toxicity
RNNs: Recurrent neural networks
VAEs: Variational autoencoders
GANs: Generative adversarial networks
MMP: Matched molecular pair
NLP: Natural language processing
VJTNN: Variational junction tree encoder-decoder
HierG2G: Hierachical graph encoder-decoder
LSTM: Long short-term memory
HLM CLint: Human Microsome Intrinsic Clearance
NLL: Negative log likelihood
RMSE: Root-mean-square error
NRMSE: Normalized RMSE
