# Supplementary material for: Molecular optimization by capturing chemist’s intuition using deep neural networks
Source: J Cheminform. 2021 Mar 20;13:26. doi: 10.1186/s13321-021-00497-0 (PMC7980633; doi:10.1186/s13321-021-00497-0)
Supplement: Supplementary file 1 — Additional file 1. Additional figures. [file 13321_2021_497_MOESM1_ESM.pdf]

Molecular Optimization by Capturing Chemist's Intuition Using Deep Neural Networks

Jiazhen He[1*], Huifang You[1,3], Emil Sandström[1,4], Eva Nittinger[2], Esben Jannik Bjerrum[1], Christian Tyrchan[2], Werngard Czechtizkyr[2], Ola Engkvist[1]

[1]Discovery Sciences, R&D, AstraZeneca, Gothenburg, Sweden
[2]Medicinal Chemistry, Research and Early Development, Respiratory and Immunology (R&I), BioPharmaceuticals R&D, AstraZeneca, Gothenburg, Sweden
[3]Department of Pharmaceutical Biosciences, Uppsala University, Uppsala, Sweden
[4]Department of Mathematics and Mathematical Statistics, Umeå University, Umeå, Sweden
[*]Correspondence: jiazhen.he@astrazeneca.com

Supplementary material

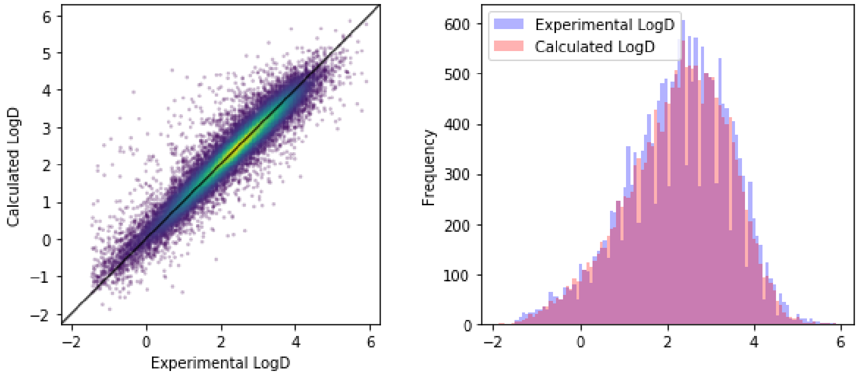

(a) Experimental LogD vs. predicted LogD and their distributions

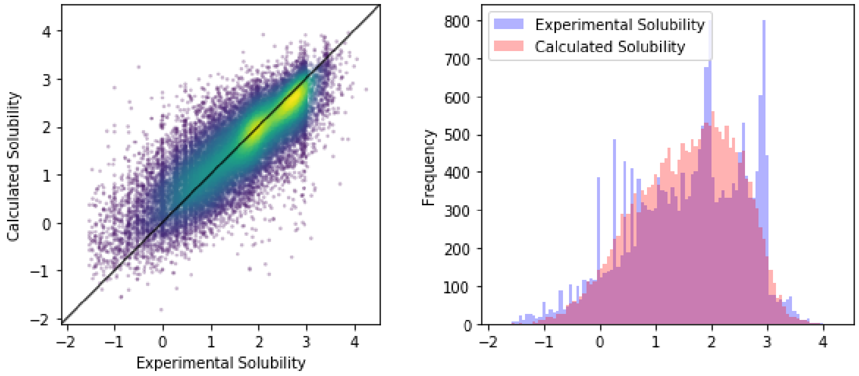

(b) Experimental solubility vs. predicted solubility and their distributions

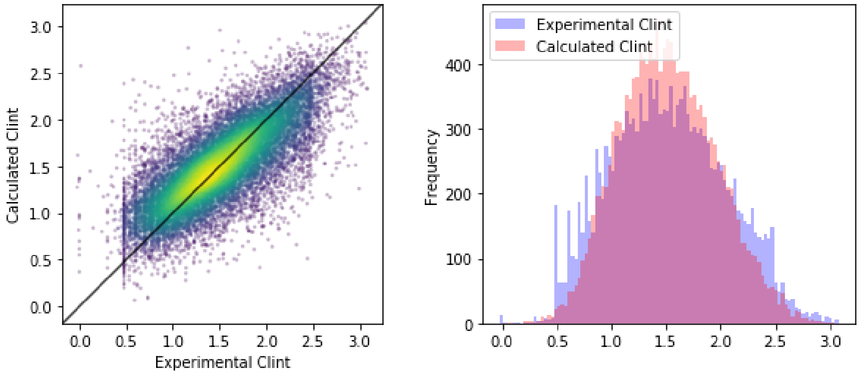

(c) Experimental clint vs. predicted clint and their distributions

Figure 1 Experimental property values vs. predicted property values and their distributions

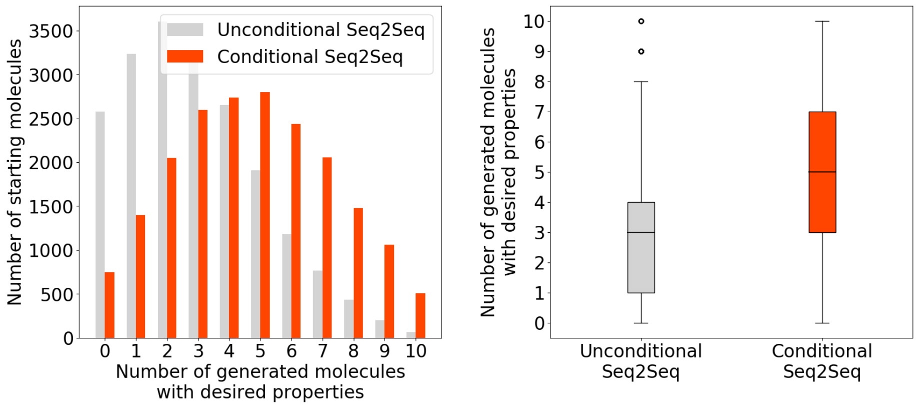

(a) Test-Original

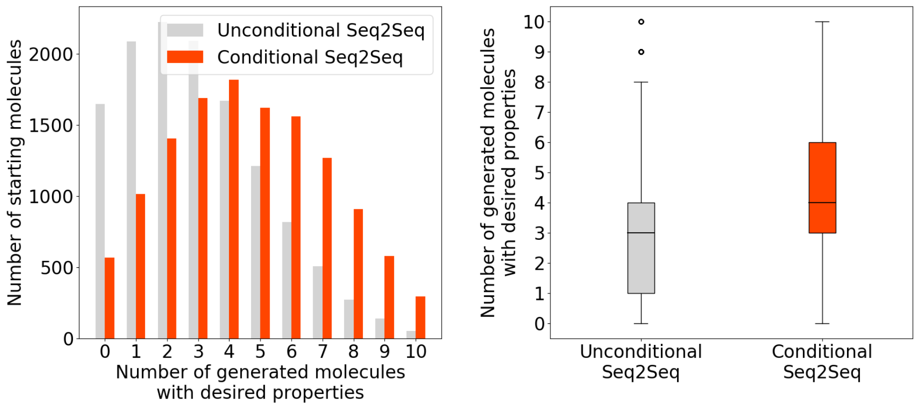

(b) Test-Molecule

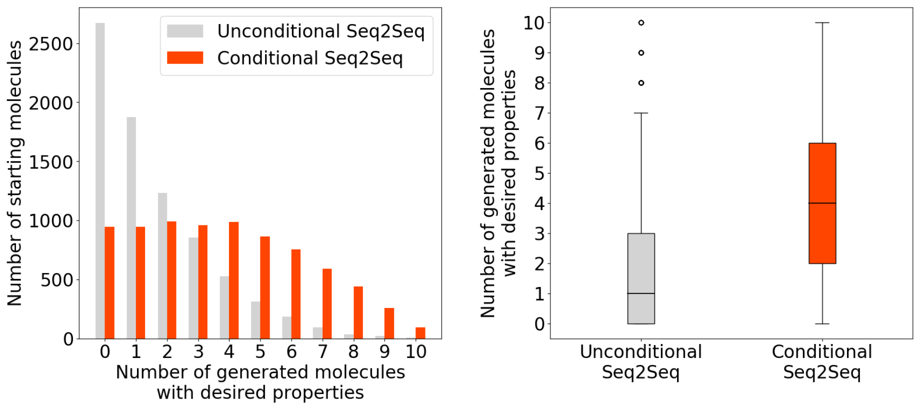

(c) Test-Property

Figure 2 Number of generated molecules with desirable properties per starting molecule using unconditional Seq2Seq and conditional Seq2Seq on three test sets. Conditional Seq2Seq outperforms unconditional Seq2Seq using K-Sample Anderson–Darling Test at significance level of 0.1%.
